# The investigation of vitamin D on the DLK/DCX pathway in cortical neuron maturation

**DOI:** 10.1002/alz70855_106349

**Published:** 2025-12-24

**Authors:** Ebru Keskin, Duygu Gezen‐Ak, Erdinc Dursun

**Affiliations:** ^1^ Cerrahpasa Faculty of Medicine, Istanbul University‐Cerrahpasa, Istanbul, Turkey; ^2^ Institute of Neurological Sciences, Istanbul University‐Cerrahpasa, Istanbul, Turkey

## Abstract

**Background:**

The dual leucine zipper kinase (DLK) signaling pathway regulates neural development aspects, including axonal growth, neuronal migration, and apoptosis, in diverse model organisms[1‐5]. Most DLK‐mutant mice exhibit perinatal mortality[6]. DLK induces JNK (c‐Jun *N*‐terminal kinase) activity in vitro. In mammals, JNK phosphorylates several target proteins, including those involved in cell movement (DCX)[7]. Studies on vitamin D treatment and primary cultures of murine neural progenitor cells have revealed an increase in DCX‐expressing cells[8]. This study, we aimed to determine the effect of vitamin D administration on the expression levels of DCX and phosphorylated DCX proteins involved in development and axon growth through the DLK‐MAPK signaling pathway in developing neurons and to identify other proteins that vitamin D may interact with.

**Method:**

10^‐8^M 1,25‐dihydroxy vitamin D3 and ethanol treatments were administered 24 h after establishing primary cortical neuron cultures from 16‐day‐old rat embryos. Protein expression levels were analyzed at 24,48,72,96,120, and 144 h post‐treatment. VDR‐immunoprecipitation (IP) was performed 30 min after treatment to illustrate the rapid response impact.

**Result:**

DLK and DCX protein expression levels significantly increased 24 hours after vitamin D treatment compared to the ethanol‐treated group (*p* <0.05 and *p* <0.001, respectively), while pDCX levels decreased significantly (*p* <0.01). At 48, 96, and 120 hours post‐administration, DLK levels remained significantly elevated in the vitamin D group compared to the control group (*p* <0.01, *p* <0.05, and *p* <0.01, respectively), but showed significant reduction at 72 and 144 hours (*p* <0.01, *p* <0.01, respectively). At 72,96, and 120 hours, DCX protein expression was significantly lower in the vitamin D group than in the control group (*p* <0.001, *p* <0.01, and *p* <0.01, respectively).pDCX protein expression levels were significantly reduced at 48, 72, and 96 hours after vitamin D treatment compared to the control group (*p* <0.05, *p* <0.01, and *p* <0.01, respectively). At 120 hours, pDCX expression was significantly higher than in the ethanol‐treated and control groups (*p* <0.05, *p* <0.05). VDR precipitated with DLK and STMN2 proteins after VDR‐Ip.

**Conclusion:**

The results of our study suggest that vitamin D administered to newly formed neurons may influence the levels of DLK, DCX, and *p*‐DCX proteins, presumably performing a regulatory role at various phases of development. VDR may interact with DLK and STMN2. This study was supported by the TÜBİTAK 1002‐A (Project No:123S438).

